# Pleomorphic Adenoma of the Palatal Minor Salivary Gland: An Unusual Case With Imaging and Cytological Correlation

**DOI:** 10.7759/cureus.94617

**Published:** 2025-10-15

**Authors:** Vamshi Krishna Reddy B, Jitendra Sharma, Hemlata Panwar, Siddhartha Banchhor, Somesh Venkataraman

**Affiliations:** 1 Radiology, All India Institute of Medical Sciences, Bhopal, Bhopal, IND; 2 Pathology and Laboratory Medicine, All India Institute of Medical Sciences, Bhopal, Bhopal, IND

**Keywords:** hard palate, intraoral tumor, minor salivary gland tumor, palatal swelling, pleomorphic adenoma

## Abstract

Pleomorphic adenoma is the most common salivary gland neoplasm, accounting for a significant proportion of both major and minor salivary gland tumors. While it most frequently arises from the parotid or submandibular glands, it can occasionally present as an intraoral mass over the palate or lip when originating from the minor salivary glands. A palatal pleomorphic adenoma typically presents as a painless, gradually enlarging mass on the posterolateral aspect of the palate. This case highlights the importance of considering pleomorphic adenoma in the differential diagnosis of palatal swellings. It also emphasizes the need for early diagnosis to prevent complications such as functional impairments or malignant transformation.

## Introduction

Pleomorphic adenoma, also known as a benign mixed tumor, is the most frequent salivary gland neoplasm, accounting for approximately 60-70% of all salivary gland tumors. While the parotid gland is most commonly affected, 10-15% of cases originate from minor salivary glands, with the palate being the predominant intraoral site [1]. The hard palate is the most frequently affected intraoral site due to the high concentration of minor salivary glands in this region. These tumors usually present as slow-growing, painless masses and are rare in children and adolescents. It is a mixed epithelial and mesenchymal neoplasm that typically presents as a slow-growing, painless, firm submucosal mass. Although benign, incomplete excision may lead to local recurrence, and, in rare cases, long-standing lesions can undergo malignant transformation. Imaging with computed tomography or magnetic resonance imaging helps define the extent of soft-tissue involvement and any associated bone remodeling or thinning [2], while fine-needle aspiration cytology provides valuable preoperative diagnostic guidance. Early recognition and complete surgical excision with adequate margins are crucial to prevent recurrence and preserve palatal function. This case report describes a 16-year-old girl with a pleomorphic adenoma of the left hard palate, highlighting the clinical, radiological, and cytological correlation essential for timely diagnosis and appropriate surgical management.

## Case presentation

**Figure 1:**
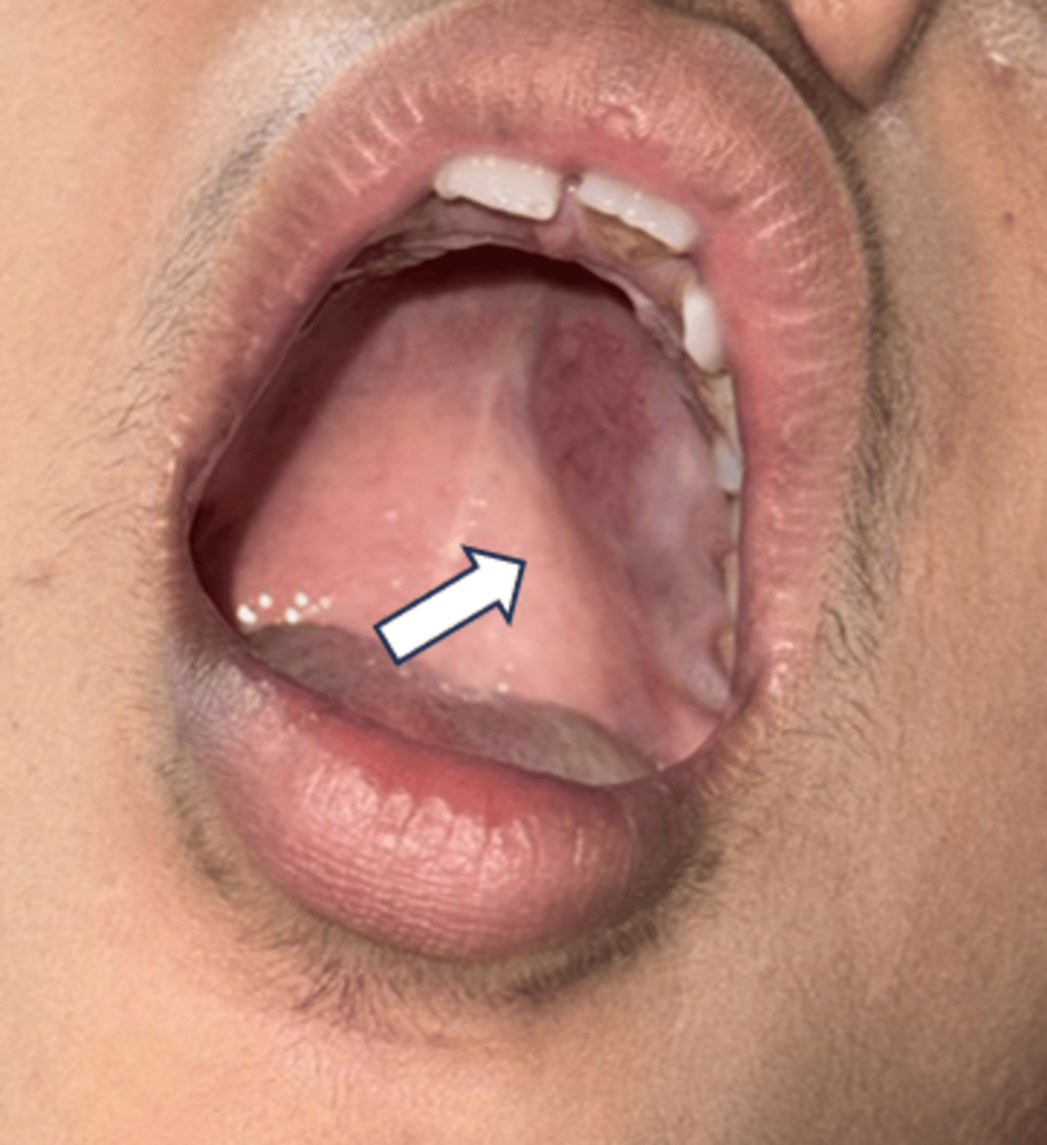
Clinical image showing a firm to hard, tender swelling over the left hard palate (white arrow), without bleeding on touch.

A 16-year-old girl presented with a two-month history of a swelling over the roof of the mouth. The swelling was not associated with pain, bleeding, trauma, infection, or similar lesions in the past. On intraoral examination, a firm-to-hard, mildly tender swelling was identified over the left hard palate. The overlying mucosa was intact with no bleeding on palpation (Figure 1).

**Figure 2:**
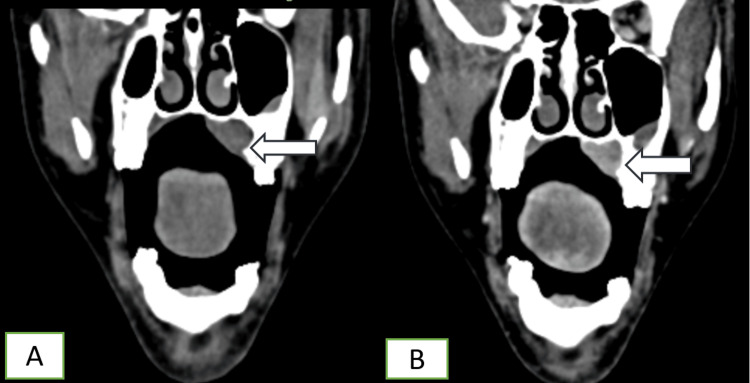
Imaging findings of a left hard palate lesion. A: NCCT coronal section of the face showing a lesion (white arrow) epicentered at the left side of the hard palate; B: lesion (white arrow) showing moderate to severe enhancement on post-contrast study. NCCT: non-contrast computed tomography

**Figure 3:**
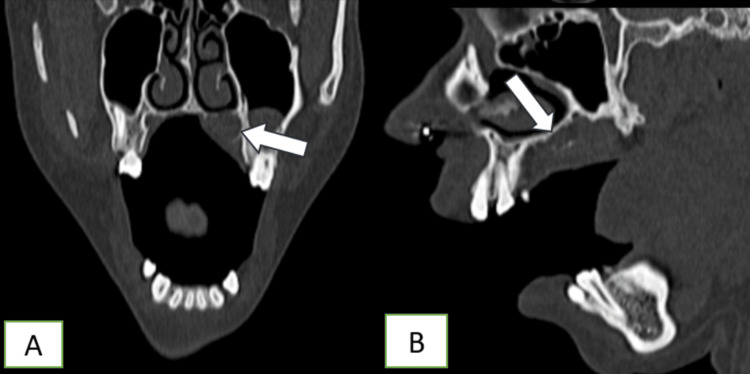
CT findings. A: CT bone window coronal; B: sagittal section showing cortical thinning and bone erosions of the involved bone (white arrows).

CT revealed a relatively well-defined, enhancing soft tissue lesion epicentered in the left side of the hard palate. The mass measured approximately 3.1 × 1.5 × 1.2 cm (anteroposterior × transverse × craniocaudal). It demonstrated soft tissue attenuation with moderate to strong enhancement on post-contrast imaging (pre-contrast 47 HU, post-contrast 137 HU). Superiorly, the lesion caused mild elevation of the hard palate, involving the floor of the left nasal cavity and the inferomedial aspect of the floor of the left maxillary sinus; the involved bone showed cortical thinning more prominent at the nasal cavity floor with small areas of interspersed erosions (Figure 2). The lesion did not cross the midline. There was also subtle thinning and scalloping of the inner cortical margins of the right upper alveolus with thin bony spurs projecting into the lesion (Figure 3). The lesion’s soft tissue merged with the tissue of the soft palate with indistinct margins.

Based on the clinical presentation and imaging findings, the lesion was suspected to be a benign minor salivary gland neoplasm, with pleomorphic adenoma being the most likely diagnosis. Its well-defined margins, slow growth, and characteristic enhancement pattern supported this impression. However, given the palatal location and the possibility of malignant transformation in salivary gland tumors, a low-grade mucoepidermoid carcinoma was considered as a close differential diagnosis. Histopathology was done for the confirmatory diagnosis.

**Figure 4:**
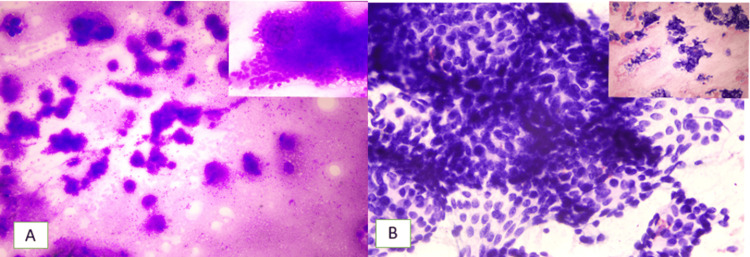
Fine-needle aspiration cytology (FNAC) findings. A: Giemsa stain, 4x; B: PAP stain, 4x. FNAC smear shows many variable-sized hyaline globules with the presence of ductal epithelial cells and myoepithelial cells embedded in a chondromyxoid stroma. Inset pictures – Higher magnification (40x) shows oval to plasmacytoid scattered ductal epithelioid and myoepitheloid cells with bland nuclear morphology. PAP stain: Papanicolaou stain

Fine-needle aspiration cytology (FNAC) of the swelling confirmed it as a pleomorphic adenoma (Milan IV-A category) (Figure 4).

Based on the clinical, radiological, and cytological findings, a diagnosis of pleomorphic adenoma arising from the minor salivary glands of the palate was established. Complete surgical excision with adequate margins was planned to achieve definitive treatment and minimize the risk of recurrence.

## Discussion

Pleomorphic adenoma of the palate typically presents as a slow-growing, painless, firm submucosal mass with intact overlying mucosa. Unlike tumors of the parotid or submandibular glands, palatal lesions often lack a true capsule, which predisposes them to local infiltration into adjacent tissues, including periosteum and, at times, underlying bone. This characteristic explains the cortical thinning and mild erosions seen in our patient [1].

Imaging plays a pivotal role in preoperative evaluation. CT is valuable in delineating soft tissue extent, bone remodeling, and erosions, while MRI offers superior contrast for assessing perineural spread and adjacent tissue involvement [2]. In our case, CT demonstrated a well-enhancing lesion centered in the left hard palate with cortical thinning and minimal erosions, features suggestive of a benign, slow-growing expansile neoplasm. FNAC remains an important adjunct for preoperative diagnosis, with our patient’s cytology confirming pleomorphic adenoma (Milan IV-A), consistent with prior literature [3].

The mainstay of treatment is complete surgical excision with adequate margins. Due to the absence of a true capsule and the risk of microscopic extensions, meticulous dissection is essential to prevent recurrence [4]. Malignant transformation, though rare, has been reported in long-standing or recurrent cases, signifying the need for timely and complete excision [5].

While pleomorphic adenomas are the most common salivary gland tumors, their occurrence in the pediatric and adolescent population is rare (<5% of cases). This rarity often leads to diagnostic delays, as other conditions such as developmental cysts, vascular malformations, or odontogenic tumors may be considered initially [6]. Awareness of this entity in younger patients is crucial, as delayed intervention may result in progressive bone remodeling, speech or masticatory dysfunction, or, rarely, malignant change [7].

Histopathologically, pleomorphic adenoma exhibits biphasic morphology with epithelial and myoepithelial components in a variable stromal background, which may appear myxoid, chondroid, or hyalinized. This histological diversity contributes to varied imaging appearances and may mimic other benign or low-grade malignant salivary tumors, particularly low-grade mucoepidermoid carcinoma and polymorphous adenocarcinoma [8]. Hence, cytological or histological confirmation is essential prior to definitive treatment [9].

The postoperative prognosis is excellent when complete excision with clear margins is achieved. Recurrence may occur if there is intraoperative tumor spillage or incomplete removal, emphasizing the need for long-term follow-up, especially in young patients, to detect recurrence or rare malignant transformation [10]. In our case, early diagnosis supported by imaging and cytological correlation enabled accurate characterization of the lesion and assisted in planning definitive surgical management.

## Conclusions

Pleomorphic adenoma of the minor salivary glands of the palate, though uncommon in adolescents, should be considered in the differential diagnosis of palatal swellings. Clinical evaluation, imaging, with cytology aid in confirming diagnosis and treatment planning. Complete surgical excision with adequate margins remains the treatment of choice, with long-term follow-up to detect recurrence.
